# Study protocol: patient reported outcomes for bladder management strategies in spinal cord injury

**DOI:** 10.1186/s12894-017-0286-3

**Published:** 2017-10-10

**Authors:** Darshan P. Patel, Sara M. Lenherr, John T. Stoffel, Sean P. Elliott, Blayne Welk, Angela P. Presson, Amitabh Jha, Jeffrey Rosenbluth, Jeremy B. Myers, for the Neurogenic Bladder Research Group

**Affiliations:** 1 Division of Urology, Department of Surgery, University of Utah Health Care, 30 North 1900 East, Room #3B420, Salt Lake City, UT 84132 USA; 2 Department of Urology, University of Michigan, Ann Arbor, Michigan USA; 3 Department of Urology, University of Minnesota, Minneapolis, Minnesota USA; 4 Divsion of Urology, University of Western Ontario, London, Ontario Canada; 5 Divsion of Epidemiology, Department of Internal Medicine, University of Utah Health Care, Salt Lake City, Utah USA; 6 Department of Physical Medicine and Rehabilitation, University of Utah Health Care, Salt Lake City, Utah USA

**Keywords:** Urinary bladder, Spinal cord injury, Patient reported outcomes, Quality of life, Incontinence, Bladder management

## Abstract

**Background:**

The majority of spinal cord injury (SCI) patients have urinary issues, such as incontinence, retention, and frequency. These problems place a significant burden on patients’ physical health and quality of life (QoL). There are a wide variety of bladder management strategies available to patients with no clear guidelines on appropriate selection. Inappropriate bladder management can cause hospitalizations and serious complications, such as urosepsis and renal failure. Patients believe that both independence and ability to carry out daily activities are just as important as physical health in selecting the right bladder-management strategy but little is known about patient’s QoL with different bladder managements. Our study’s aim is to assess patient reported QoL measures with various bladder managements after SCI. This manuscript describes the approach, study design and common data elements for our central study.

**Methods:**

This is a multi-institutional prospective cohort study comparing three different bladder-management strategies (clean intermittent catheterization, indwelling catheters, and surgery). Information collected from participants includes demographics, past medical and surgical history, injury characteristics, current and past bladder management, and SCI /bladder-related complications. Patient reported outcomes and QoL questionnaires were administered at enrollment and every 3 months for 1 year. Aims of this study protocol are: (1) to assess baseline QoL differences between the three different bladder-management strategies; (2) determine QoL impact when those using either form of catheter management undergo a surgery over the 1 year of follow-up among patients eligible for surgery; (3) assess the effects of changes in bladder management and complications on QoL over a 1-year longitudinal follow-up.

**Discussion:**

By providing information about patient-reported outcomes associated with different bladder management strategies after SCI, and the impact of bladder management changes and complications on QoL, this study will provide essential information for shared decision-making and guide future investigation.

**Trial registration:**

Trial registration number: www.clinicaltrials.gov: Identifier: NCT0261608; U.S. National Library of Medicine, wwwcf.nlm.nih.gov: Identifier: HSRP20153564.

## Background

Nearly 250,000 Americans live with a spinal cord injury (SCI) [1]. Approximately 74-80% of SCI individuals report some degree of bladder dysfunction within 1 year of injury [2–4]. Urinary dysfunction has a significant clinical, physical, and quality of life (QoL) burden in patients with SCI and neurogenic bladder. Common urinary symptoms, after SCI, include urinary retention, incontinence, and increased urinary frequency / urgency. Inappropriate bladder management can cause significant complications, including recurrent urinary tract infections (UTIs), urosepsis, and progressive renal failure due to high pressures within the bladder [5]. In addition to bladder dysfunction, SCI patients usually have physical limitations due to their injury, which may have a compound negative effect on their QoL [6, 7].

Multiple bladder management options are available for urinary issues in SCI patients including medications to reduce bladder spasticity and pressure, catheter based management (intermittent or permanent indwelling catheters), and reconstructive surgery to expand the bladder or bypass the bladder via a conduit or continent catheterizable pouch urinary diversion. The gold standard for management of bladder dysfunction includes clean intermittent catheterization (CIC) to empty the bladder (passing a temporary catheter periodically during the course of a day) and/or medications to reduce bladder spasticity and pressure. This strategy can significantly reduce urinary complications and related hospitalizations in patients who are able to tolerate the medications and perform urethral catheterizations. However, despite the well-recognized benefits to CIC, only 30% of SCI individuals who start CIC continue it over time and mostly patients transition to indwelling catheters (IDC), which is the management strategy with the highest complication rate [8–10]. The underlying reasons for this transition over time are not known but likely rooted in QoL issues, such as inconvenience, dependence on others, privacy, and incontinence between catheterizations [4, 11].

Most studies evaluating treatment options for neurogenic bladder focus on clinical outcome measures, such as the number of incontinent episodes in a day or the number of urinary tract infections in a year. The impact of these clinical parameters on patient-centered outcomes such as QoL is unclear. Additionally, there are some disparities in clinical and patient-centered outcomes for different bladder management strategies with some studies indicating a preference for IDC over CIC [8, 12]. Bladder management affects an individual’s daily experience with SCI and is the second most common topic that SCI patients want to address when seen by a healthcare provider. Therefore, in order to provide more patient-centered care, it is critical to define patient reported outcomes and QoL in order to target issues that are important to patients in future studies.

One of the greatest knowledge gaps for bladder management strategies in SCI exists for reconstructive bladder surgery, such as bladder augmentation cystoplasty or urinary diversion. In our review of the literature, there were only 4 studies with a median sample size of 21 that assessed patient reported outcomes using non-validated instruments following urinary diversion in neurogenic bladder patients [13]. Closing this knowledge gap is important because these procedures are major surgeries and performed often in patients with sub-optimal medical health. In addition to deficits in patient reported outcomes with different bladder managements, there is also very little known about how complications, such as those associated with indwelling catheters, adversely affect patient QoL. Most published studies are cross-sectional or measure QoL before and after an intervention with little long-term follow-up.

The primary aim (*aim 1*) of our study it to determine baseline patient reported QoL with three different bladder management strategies (CIC, IDC, and surgery) in SCI. The secondary aim (*aim 2*) is to assess the comparative effectiveness of CIC/IDC versus surgery in terms of QoL over the 1 year of follow-up among patients eligible for surgery. The tertiary aim (*aim 3*) of our study is to determine, during longitudinal follow-up, how changes in bladder management and urinary tract complications affect patient reported outcomes and QoL. The overall goal of the study is to gain critical knowledge in this area to facilitate shared decision making surrounding bladder management after SCI and guide future patient-centered investigation.

## Methods/Design

### Study design

This study is a multi-institutional prospective cohort study of patient reported outcomes of adult patients with acquired SCI comparing 3 different bladder management methods (CIC, IDC, and reconstructive surgery).

### Study location

The three study locations are the Universities of Michigan, Minnesota, and Utah, which all have dedicated SCI treatment centers and urologic specialty clinics for neurogenic bladder management. Institutional review board approval was obtained from each study site before beginning the study.

### Study population and recruitment

Inclusion and exclusion criteria are listed in Table 1. To meet eligibility criteria, a patient must have SCI and neurogenic lower urinary tract symptoms. Study inclusion is also limited to patients with acquired etiologies of SCI, such as trauma, tumor without current progressive malignancy, spinal cord stroke, vascular bleed such as an arterio-venous malformation, transverse myelitis without progression to multiple sclerosis, and post-surgical / procedural complications. Any level of SCI, including those with cauda equina, are eligible to participate in the study. Exclusion criteria are progressive spinal disorders (e.g. multiple sclerosis, active malignancy, other progressive neurologic or neuromuscular diseases), as well as congenital forms of SCI (myelomeningocele, spina bifida, cerebral palsy, etc.).

**Table 1:** Study inclusion and exclusion criteria for patients with spinal cord injury

Inclusion criteria:	
• Age ≥ 18 years • Ability to effectively communicate in English • Ability to provide informed consent • Willing to participate and answer 5 sets of questionnaires over 1-year. • Acquired SCI – traumatic, spinal cord stroke, malignancy (not active), surgical injury, transverse myelitis.	
Exclusion criteria:	
• Congenital SCI – cerebral palsy, spina bifida, caudal regression, sacral agenesis. • Progressive SCI – multiple sclerosis, active malignancy, progressive neurologic diseases leading to SCI	

A priori recruitment goals based on power calculations was 900-1300 total participants. Participants will be recruited over a 1-year period and followed for an additional year. Recruitment will occur at multiple settings for each university. These settings include: urology clinics, physical medicine and rehabilitation SCI clinics, physical therapy centers, inpatient rehabilitation hospital, and chronic SCI residential facilities. Other settings for local recruitment include SCI-oriented informational seminars, and other SCI-oriented activities. In addition to local in-person recruitment, the study will be available for remote enrollment via a web-based portal. Participants in the United States and Canada may fill out a screening eligibility form on a website created for promotion of bladder research and information (NBRG.org). If eligible, interested participants will be contacted by a research coordinator from 1 of the 3 study centers. The study will be advertised via social media (Twitter and Facebook) and on SCI advocacy groups websites. In addition, experts in the field on neurogenic bladder will be contacted and asked to advertise and promote the study to patients they see in a clinical setting.

### Patients as research team participants

One key to patient-centered research is engagement of patient and clinical “stakeholders.” Stakeholders are individuals with personal and or professional insight into the disease process or interventions being studied. This study involves stakeholders in all aspects of the research process in order to assure that the research design, implementation, and dissemination of the results are relevant to clinicians, patients, and caregivers dealing with the SCI on a day-to-day basis. We will assemble a team of people living with SCI to create a Patient Advisory Group, which will provide input regarding the following parameters: meaningfulness of research questions, important characteristics of study participants, comparators, major outcomes, monitor the study progress, ease of questionnaire administration, advocate change in study design where needed, and suggest implementation plans for dissemination of relevant findings.

### Study aims

#### Aim 1

To compare cross-sectional patient reported outcome measures and QoL for 3 different bladder management methods (1) CIC, (2) IDC, and (3) reconstructive surgery. We will use the two bladder-specific instruments from the Spinal Cord Injury Quality of Life (SCI-QoL) panel and Neurogenic Bladder Symptom Score (NBSS) for this purpose.

#### Aim 2

To determine the comparative effectiveness of CIC/IDC versus surgery in terms of QoL (using the two bladder-specific SCI-QoL instruments and the NBSS) over 1-year of follow-up. This analysis will be conducted in a causal inference framework among the patients who have not previously had surgery but are eligible for surgery.

#### Aim 3

To measure the effect of bladder management changes and urinary complications on patient reported bladder management outcomes and QoL (using the two bladder-specific SCI-QoL instruments and the NBSS). Longitudinal collection of these instruments will allow analysis of participants who have either of these events during the course of the study.

### Study procedures

**Fig. 1:**
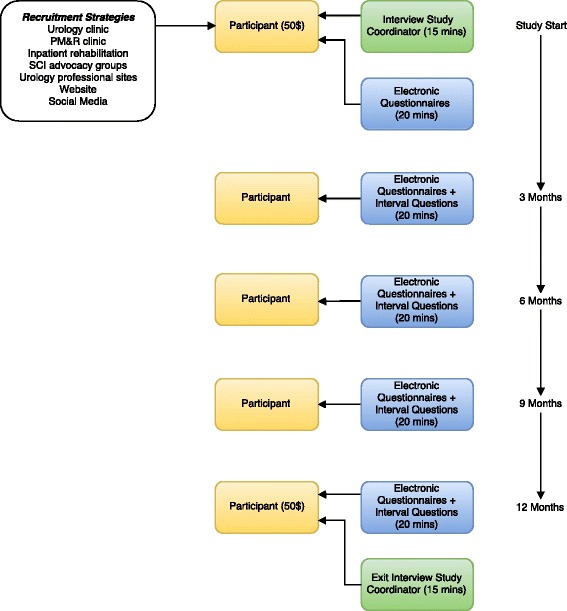
Summary of study procedures

The study procedures are summarized in Fig. 1. Upon enrollment participants will undergo an interview with a trained study coordinator gathering information including demographics, SCI injury specifics, pertinent past medical and surgical history, past and current bladder management, SCI-related complications, and urologic follow-up over time. If acute rehabilitation location is known, records will be obtained and SCI injury specifics and management will be confirmed. After the enrollment interview participants will answer a panel of questionnaires designed to assess many aspects of patient reported bladder outcomes and QoL. These questionnaires were deemed relevant by investigators and the Patient Advisory Panel. Every three months for the next 12 months after enrollment, the same panel of questions will be electronically sent to the participants, along with non-validated questions about changes in bladder management or urologic complications occurring in the intervening 3 months.

**Fig. 2:**
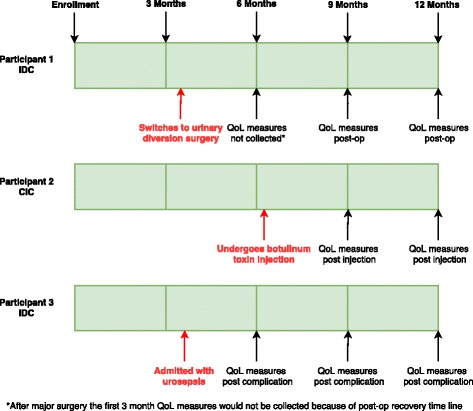
Patient changes in management or significant treatments and complications and how QoL is captured post these events

Longitudinal follow-up will occur for 12 months. A subject can potentially switch bladder-management strategy group during the course of the follow-up or experience a urologic complication. Figure 2 illustrates possible scenarios using 3 example patients. QoL data will not be collected within 3 months of a major surgery (bladder augmentation, urinary diversion), however, other interventions such as first-time Onabotulinum toxin A injection will be assessed at the next scheduled 3-month interval after the intervention. Urinary complications will be assessed in a similar fashion. These complications will include but are not limited to: admission for UTI/pyelonephritis, urinary stone episode, any urological complication (orchitis, gross hematuria, etc.), need for surgical treatment to treat complications of previous surgery. We estimate that switching bladder-management strategy (for example, participant 1 in Fig. 2) could happen for approximately 10-15% of patients based upon the experience of the research team.

After the enrollment interview, patients will complete the panel of questionnaires on an electronic tablet or via a web-based platform accessible from any location with internet access. A study coordinator will be available if they need assistance with electronic questionnaire completion either in person or over the phone. All electronic questionnaires will be administered via the secure and Health Insurance Portability and Accountability Act (HIPAA)-compliant Assessment Center^SM^ platform (www.assessmentcenter.net). This online data collection portal supports repeated administrations of the same questionnaires at pre-determined time points, real-time monitoring of questionnaire completion and data integrity analysis. Participants will be electronically prompted to complete their next due questionnaires 15-days prior to their next 3-month questionnaire due-date and for 15-days afterwards. Research coordinators will routinely monitor completion and those that are near past-due will be sent e-mail and/or phone reminders requesting their completion of the set of questionnaires to improve adherence. With successful completion of the first panel of questionnaires, participants will be provided with $50 gift card to the preferred retailor of their choice (e.g. Amazon, Walmart, etc.). An additional $50 gift card will be given to participants after completion of 1 year of follow-up questionnaires. As mentioned above, at each 3-month questionnaire administration, participants will also receive non-validated questions about interval changes in their bladder management or urologic complications. Changes and complications will therefore be tracked over time. Clarifications needed based on these interval responses will be followed-up with a phone call from a research assistant. In addition, at the end of 12-months a telephone administered exit interview with a research assistant will be performed which will confirm if there are any additional changes in bladder management or urinary complications over the course of the study. A Likert 15-point scale will be given to participants assessing the impact from these ‘events’ over the course of the year from “a very great deal worse” to “a very great deal better”.

### Outcome instruments

A full list of patient-reported outcomes and QoL measures is summarized in Table 2.

**Table 2:** Patient reported outcome measures used for the study

Bladder specific:	
• Neurogenic Bladder Symptom Score	
• SCI-QoL	
○ Bladder management difficulties	
○ Bladder complications	
General QoL:	
• Modified SF-12	
Psychosocial:	
• SCI-QoL	
○ Pain interference	
○ Independence	
○ Positive affect and well-being	
○ Satisfaction with social roles and activities	
Other:	
• Likert pain scale	
• Autonomic dysreflexia	
• SCI-QoL	
○ Basic mobility	
○ Fine motor	
○ Self-care	
• Bowel function	
○ Neurogenic Bowel Dysfunction Score	
○ SCI-QoL – Bowel management difficulties	

#### Bladder-specific measurements

The bladder-specific instruments from the SCI-QoL measurement system and the NBSS are the outcome measures used for all aims of this study. The SCI-QoL measurement system is a validated, comprehensive patient-reported outcome measurement item bank panel, which consists of 19 item banks including two item banks related to complications and consequences of bladder management (“Bladder Complications”), as well as feelings about bladder related limitations and function (“Bladder Management Difficulties”) [14]. SCI-QoL instruments uses item response theory and computerized adaptive testing using the Assessment Center^SM^ platform. Computer adaptive testing utilizes participant responses to guide the administration of only select, pertinent questions drawn from a larger item bank. For example, when chronically ill patients with limited functioning responds that they can’t get out of bed without help, they are not next asked if they can jog a mile. Instead the participant is asked more narrowed questions relevant to their mobility level, such as “Can you brush your hair or open a jar?” A final calibrated score is produced for each health domain to provide an individual’s QoL or function score. Computer adaptive testing minimizes floor and ceiling effects and allows accurate assessment over a wide range of function and symptoms while minimizing participant burden and maximizing patient relevance.

The NBSS is a 24-item validated instrument with three domains, as well as one question about global satisfaction with urinary function. The three domains of the NBSS are incontinence, storage and voiding, and urinary complications. The NBSS was developed and validated in a diverse group of neurogenic bladder patients, including those with SCI, multiple sclerosis, and congenital neurogenic bladder. This instrument incorporates self-reported urinary complications such as urinary tract infections, kidney and bladder stones, and pain associated with urination or catheter use. The questionnaire emphasizes function and consequences rather than feelings and social limitations.

#### General QoL measurements

The Short Form-12 (SF-12) from the Medical Outcomes Study will be used to asses general QoL. The SF-12 is a generic QoL instrument used very commonly and has been modified for people that utilize wheelchairs and have SCI [15]. There is considerable population-level normative data using the SF-12 that is available for comparison with study populations of interests (39, 40).

#### Psychosocial measurements

Patient Advisory Group and other clinical stakeholders provided guidance on selection of other measures of non-bladder related psychosocial functioning, which we thought would potentially influence how participants would report their bladder function. For example, we postulated that a person who was depressed with SCI might report much worse feelings about bladder function than someone who was not depressed. The final selected psychosocial measures encompass the following areas: independence, pain interference with life, satisfaction with social participation and roles, and positive affect and well-being [16]. These instruments are all SCI-QoL item banks and administered with computer adaptive testing via the Assessment Center^SM^ platform.

#### Other measurements of physical function

Spinal cord injury affects many other aspects of physical function and all of these in turn have the potential for affecting how a patient reports their bladder function and related QoL. For instance, if a patient has severe bowel dysfunction they may also report worse bladder dysfunction. In an effort to capture other physical limitations and dysfunction associated with SCI and evaluate the relationship to bladder satisfaction, we asked participants about a variety of other physical functioning. These areas of physical function included: autonomic dysreflexia, pain level, ability for self-care, mobility, fine motor function, and bowel dysfunction. These questionnaires include (1) autonomic dysreflexia questions based on a longer validated Autonomic Dysfunction Following Spinal Cord Injury (ADFSCI) instrument [17], (2) a 0-10 point Likert pain scale, (3) the validated Neurogenic Bowel Dysfunction Score (NBD) [18], (4) three functional–index physical function banks from SCI-QoL (Basic mobility, Fine motor, Self-care), [14, 19], and (5) the SCI-QOL bowel management difficulties computer adaptive item bank.

### Statistical analyses and outcomes

#### Primary outcome

The primary outcomes are the total scores from the following instruments: SCI-QoL Bladder Management Difficulties, SCI-QoL Bladder Complications, and the NBSS. These will be compared between different management groups (CIC, IDC, and reconstructive surgery) in *aim 1*, between CIC/IDC versus surgery among those eligible for surgery in *aim 2*, and before and after changes in bladder management or complications in *aim 3.* Non-eligible candidates for surgery include patients whose risk of an unfavorable outcome is higher than average.

#### Secondary outcomes

The secondary outcomes include the NBSS subdomains of (1) Incontinence, (2) Storage and voiding, (3) Complications, and the NBSS single global question about satisfaction with urinary system function. Secondary outcomes will undergo the same comparisons and modeling frameworks used for primary outcomes*.*

##### Aim 1

To address the question comparing QoL related to CIC, IDC and surgery at baseline, we will compare bladder management strategy with our primary and secondary outcomes in both univariable and multivariable linear regression models. Multivariable models will adjust for age, sex, years since injury, complete injury, pain, education level, and severity of bowel dysfunction. Models will be constructed within paraplegic and tetraplegic patients, as the relationship between QoL and bladder management strategy differs across these injury types.

##### Aim 2

To address Aim 2, comparing the effectiveness of CIC/IDC bladder management with surgery in terms of longitudinally collected QoL data, we first subset our data to patients that could theoretically be randomized to either maintaining CIC/IDC bladder management or receiving surgical treatment. Thus we will exclude patients who have previously had surgery. We plan to estimate the causal effect of surgical treatment at any point during the 1-year assessment period of our study compared to continuation of CIC/IDC throughout this period. Because the treatment will be administered at different times for different patients, and because confounding will be time dependent, standard methods using regression analysis or propensity scores for treatments at a fixed time are not applicable. Instead, we will apply inverse probability weighting under a marginal structural model (MSM) to evaluate the comparative effectiveness of surgery versus CIC/IDC among patients who are candidates for surgery. Specifically, we will compare mean levels of patient outcomes at the final 12-month time point between patients who have received surgery during the 1 year assessment period versus patients who continued CIC/IDC management, controlling for time-dependent confounding by using stabilized weights. The main time-dependent confounders include complications and SCI-QoL and NBSS – as these variables may indicate a need or desire for surgery, be influenced by surgery, and potentially affect patient-reported outcomes at the final time point. The final stabilized weights are the product of stabilized inverse probability of treatment weights (IPTW), which account for confounding, and inverse probability of censored weights (IPCW), which account for loss-to-follow-up [20]. The product of IPTW and IPCW is the subject-specific weight used in the MSM to adjust for time-dependent confounding. Applying the stabilized weights essentially generates a pseudo-population under which subjects are randomly assigned to treatment and have complete follow-up information under the assumption that the measured covariates fully account for confounding. In this pseudo-population we will estimate the average causal effect of surgical treatment during the 1-year study period using regression analyses with an indicator variable for surgery during the study period as the predictor variable while adjusting for the baseline covariates. We will use this model to report the comparative effectiveness of surgery in terms of the average difference in SCI-QoL and NBSS scores between the two groups, along with 95% confidence intervals. Additional analyses will estimate the average causal effect of receiving the surgical treatment at varying times prior to the 12-month outcome assessment.

We will plan to report the covariate summaries for the CIC/IDC and surgery groups at baseline and at each follow-up time point (3, 6, 9, and 12 months) for the time-varying measures. Fixed-time covariates will include: age, sex, years since injury at baseline, complete injury, pain, education level, and severity of bowel dysfunction. Time-varying covariates will include complications, SCI-QoL, NBSS, and the measurement time point. Complications will be included in the stabilized weights at all time points (baseline, 3, 6, 9, and 12 months). SCI-QoL and NBSS will be included at all time points < 12 m, and they will serve as outcomes (in their respective MSMs) at the final 12-month time point.

Multiple imputation will be used to impute missing data. Baseline and follow-up factors beyond the variables being analyzed will be incorporated into the imputation model to account for dependence of the missing data mechanism on other measured factors. We will apply the method of data augmentation using Markov Chain Monte Carlo (MCMC) to generate imputed values [21, 22].

##### Aim 3

To assess the effects of changes in bladder management strategy and complications on QoL, we will take a descriptive approach due to the potentially limited sample size. We expect that 10-15% of patients will change bladder management strategy. Among those who change bladder management strategy, we will compare the impact of this change on QoL outcomes using linear mixed effects models with an indicator for first/s bladder management strategy, controlling for a few key covariates (age, sex, time since injury, complete/incomplete injury and parapalegic/tetrapalegic). Correlation of the QoL outcomes within subjects will be modeled with a compound symmetry covariance matrix. We will use a similar model framework to examine the effect of complications on QoL, where this analysis will be conducted within subjects who experienced a complication. We will test whether QoL differed before and after the complication occurred by including an indicator for first/s bladder management strategy, and controlling for the same key covariates.

### Determination of sample size

We designed our study to detect clinically important differences in QoL between the 3 bladder management strategy groups (1) CIC, (2) IDC, and (3) reconstructive surgery. Practically, we anticipated the ability to recruit approximately 900-1350 subjects over a 1-year time frame after accounting for a 10% loss to follow-up. We expected CIC to comprise about 24% of our sample, IDC to be about 41%, and reconstructive surgery to be about 34%. The primary bladder specific patient-reported outcome measures used to compare these groups are the two bladder-specific SCI-QoL instruments (Bladder Complications and Bladder Management Difficulties) and the NBSS. Our effect size calculations were based on a mixed models analysis of repeated measures data assuming a conservative correlation of 0.8 and an autoregressive order 1 AR(1) covariance structure between five repeat measures on the same subject. Assuming a standard deviation of 10 for both SCI-QoL and NBSS based on previous studies and a mean of 50 and 20 respectively, we would have 80% power at a Bonferroni-adjusted alpha level (0.05/3 = 0.017, for three pair-wise comparisons among our bladder management strategies) to detect the minimum differences presented in Table 3 [23, 24]. For the SCI-QoL bladder-specific instruments, we expect to detect differences in the overall score of about 4-5% or 3-4% for our minimum and maximum sample sizes, respectively. For the NBSS, we expect to detect score differences of about 10-12% or 8.5-10% for our minimum and maximum sample sizes, respectively.

**Table 3:** Estimates of number of participants to detect differences in the SCI-QoL and the NBSS

Questionnaire	Comparison groups	Detected differences *n* = 900	Detected differences *n* = 1350
SCI-QoL	IDC vs CIC	2.34 (4.7%)	1.91 (3.8%)
	IDC vs. surgery	2.11 (4.2%)	1.72 (3.4%)
	CIC vs. surgery	2.42 (4.8%)	1.98 (4.0%)
NBSS	IDC vs CIC	2.34 (11.7%)	1.91 (9.6%)
	IDC vs. surgery	2.11(10.6%)	1.72 (8.6%)
	CIC vs. surgery	2.42(12.1%)	1.98 (9.9%)

*SCI-QoL* Spinal Cord Injury Quality of Life Scale, *NBSS* Neurogenic Bladder Symptom Score, *CIC* clean intermittent catheterization, *IDC* indwelling catheter

### Ethics and dissemination

The following precautions will be taken to ensure subject privacy is protected: research and questionnaire completion will be conducted in private place; all discussion regarding involvement in the study and study related data collection will occur in private; collected information about participants will be limited to the amount necessary to achieve the aims of the study; no unneeded information will be collected or stored. This study is being performed in accordance with the World Medical Association Declaration of Helsinki and after approval from each sponsoring center’s Institutional Review Boards [25]. This study has been registered at clinicaltrails.gov (https://clinicaltrials.gov/ct2/show/NCT02616081).

We plan to use several strategies to enhance rapid dissemination and implementation of our findings to improve patient-centered healthcare delivery and shared decision making for those with SCI. We have developed a project resource website founded by our research group, the Neurogenic Bladder Research Group (www.NBRG.org). The website is available for patient recruitment and will be used for rapid dissemination of findings. Throughout duration of the study, clinicians and patient partners will blog about their personal experience being involved in the project and provide research progress updates. Social media sites such Twitter© and Facebook© will be used to increase traffic to our website and bring global interest to our study and subsequent findings. This will be an integral aspect of the dissemination of the final results of our research. Once results for the study are available, an informational web special will be available on the (www.NBRG.org) website explaining the relevant findings and implications for patients, caregivers, clinicians, and researchers. Additionally, study participants will be emailed a link to this web special to review relevant findings in this study as a result of their participation. For clinicians and other stakeholders that follow the peer-reviewed literature, we will publish our findings in a diverse number of journals to reach urologists and physical medicine & rehabilitation specialists that would be interested in our findings.

## Discussion

Our study will address critical knowledge gaps in patient-centered outcomes for various bladder management strategies in SCI patients. This study is multi-centered, drawing participants from three large academic referral centers for SCI and complex urologic care. The use of open enrollment via the internet will augment our recruitment goals and help improve the generalizability of our findings. Additionally, our use of robust, validated instruments for assessment of patient reported outcomes and health related QoL strengthens our study. We believe that information about patient reported outcomes and QoL with common bladder management strategies after SCI, as well as the longitudinal collection of patient-reported outcomes with changes in bladder management and urinary specific complications will advance patient-centered care and shared decision-making.

### Trial status

The trial is in the recruiting phase at the time of manuscript submission.

## Abbreviations

CI: Confidence interval
CIC: Clean intermittent catheterization
IDC: Indwelling catheter
NBSS: Neurogenic Bladder Symptom Score
QoL: Quality of life
SCI: Spinal cord injury
SCI-QoL: Spinal cord injury-quality of life questionnaire
SF-12: Short form 12
UTI: Urinary tract infection
